# Characteristics and Prognostic Factors of Pulmonary Fibrosis After COVID-19 Pneumonia

**DOI:** 10.3389/fmed.2021.823600

**Published:** 2022-01-31

**Authors:** Elisabetta Cocconcelli, Nicol Bernardinello, Chiara Giraudo, Gioele Castelli, Adelaide Giorgino, Davide Leoni, Simone Petrarulo, Anna Ferrari, Marina Saetta, Annamaria Cattelan, Paolo Spagnolo, Elisabetta Balestro

**Affiliations:** ^1^Respiratory Disease Unit, Department of Cardiac, Thoracic, Vascular Sciences and Public Health, University of Padova and Padova City Hospital, Padova, Italy; ^2^Department of Medicine, Institute of Radiology, University of Padova and Padova City Hospital, Padova, Italy; ^3^Division of Infectious and Tropical Diseases, University of Padova and Padova City Hospital, Padova, Italy

**Keywords:** SARS-CoV-2, coronavirus disease 2019, pulmonary fibrosis, high-resolution computed tomography, pulmonary sequelae

## Abstract

**Background:**

Few is known about the long-term pulmonary sequelae after COVID-19 infection. Hence, the aim of this study is to characterize patients with persisting pulmonary sequelae at follow-up after hospitalization. We also aimed to explore clinical and radiological predictors of pulmonary fibrosis following COVID-19.

**Methods:**

Two hundred and 20 consecutive patients were evaluated at 3–6 months after discharge with high-resolution computed tomography (HRCT) and categorized as recovered (REC) or not recovered (NOT-REC). Both HRCTs at hospitalization (HRCT_0_), when available, and HRCT_1_ during follow-up were analyzed semiquantitatively as follows: ground-glass opacities (alveolar score, AS), consolidations (CONS), and reticulations (interstitial score, IS).

**Results:**

A total of 175/220 (80%) patients showed disease resolution at their initial radiological evaluation following discharge. NOT-REC patients (45/220; 20%) were mostly older men [66 (35–85) years vs. 56 (19–87); *p* = 0.03] with a longer in-hospital stay [16 (0–75) vs. 8 (1–52) days; *p* < 0.0001], and lower P/F at admission [233 (40–424) vs. 318 (33–543); *p* = 0.04]. Moreover, NOT-REC patients presented, at hospital admission, higher ALV [14 (0.0–62.0) vs. 4.4 (0.0–44.0); *p* = 0.0005], CONS [1.9 (0.0–26.0) vs. 0.4 (0.0–18.0); *p* = 0.0064], and IS [11.5 (0.0– 29.0) vs. 0.0 (0.0–22.0); *p* < 0.0001] compared to REC patients. On multivariate analysis, the presence of CONS and IS at HRCT_0_ was independent predictors of radiological sequelae at follow-up [OR 14.87 (95% CI: 1.25–175.8; *p* = 0.03) and 28.9 (95% CI: 2.17–386.6; *p* = 0.01, respectively)].

**Conclusions:**

In our population, only twenty percent of patients showed persistent lung abnormalities at 6 months after hospitalization for COVID-19 pneumonia. These patients are predominantly older men with longer hospital stay. The presence of reticulations and consolidation on HRCT at hospital admission predicts the persistence of radiological abnormalities during follow-up.

## Background

Coronavirus disease 2019 (COVID-19), which is caused by the severe acute respiratory syndrome coronavirus 2 (SARS-CoV-2), has infected more than 130 million people worldwide. COVID-19 leads to respiratory manifestations that can range from mild flu-like symptoms such as fever, cough, and fatigue to severe respiratory failure requiring intensive care (1, 2).

Data from previous pandemics caused by coronaviruses suggested that there may be pulmonary sequelae in one-third of patients at 12 weeks after discharge (3, 4).

Some recent studies tried to characterize radiological sequelae after COVID-19 pneumonia (5, 6). This condition, which is referred to as “post-COVID syndrome,” still lacks a universally agreed definition (7). On May 2020, a document of the British Thoracic Society (BTS) proposed an algorithm on post-discharge management of patients with COVID-19 and distinguished two groups of interest: patients with severe pneumonia and patients with mild-to-moderate pneumonia (8). Following up on this document, George and colleagues suggested a structured respiratory follow-up for patients with clinico-radiological confirmation of COVID-19 pneumonia (9). Importantly, they proposed patients with severe pneumonia undergo a full clinical assessment at 12 weeks with a chest X-ray whereas patients with persisting radiological abnormalities should undergo a high-resolution computed tomography (HRCT) scan. In this regard, the role of chest X-ray and HRCT in disease management both during hospitalization and follow-up is well established (10, 11). Han and coauthors recently reported that fibrotic-like changes on CT performed at 6 months during follow-up persist in approximately one-third of patients with COVID-19 (12), but the data on long-term pulmonary sequelae in this patient population remain scarce. The aim of this study is to characterize, among patients hospitalized for COVID-19 pneumonia, those presenting persisting pulmonary sequelae during follow-up, and to define which clinical and radiological features are predictive of persistent radiological abnormalities.

## Materials and Methods

### Study Population and Study Design

We prospectively collected patients evaluated at the post-COVID clinic of the University Hospital of Padova between June and December 2020. The patients evaluated at the post-COVID clinic were initially admitted to the Division of Infectious and Tropical Diseases of the University Hospital of Padova between February and September 2020 for SARS-CoV-2 infection confirmed by the real-time polymerase chain reaction (RT-PCR) at nasopharyngeal swab.

Among all patients evaluated, we specifically followed up every 3 months those presenting a COVID-19-related severe disease according to the WHO criteria (*n* = 220) (13). Demographics and clinical data at hospital admission [symptoms, gas exchange values (paO_2_/FiO_2_)] and during hospitalization [days of hospital stay, maximal FiO_2_ (FiO_2_ max) needed, level of care, treatment] were collected. Comorbidities were categorized as cardiovascular diseases (CVDs), respiratory diseases, metabolic diseases (including diabetes mellitus, obesity, and dyslipidemia), autoimmune diseases, and oncologic diseases (including lung, prostate, pancreatic, breast, and colon cancer). Based on patient's clinical conditions during hospitalization, we distinguished those requiring a low- (LIMC) and high-intensity medical care (HIMC), as previously described (14).

### Radiological Evaluation

At follow-up, HRCT was available for the entire study population (HRCT_1_) whereas at hospital admission, it was available in only a subgroup of patients (HRCT_0_) (*n* = 79, 36%). The HRCTs were performed by a 64 slice Siemens Somatom Sensation (Siemens Healthcare, Erlangen, Germany) applying a slice thickness ≤0.5 mm.

According to the presence or absence of radiological abnormalities on HCRT_1_, the study population was categorized as recovered patients (REC, *n* = 175) or not recovered patients (NOT-REC, *n* = 45).

Two expert thoracic radiologists (CG and AG), who were blinded to clinical data and timing of HRCTs, scored the images independently using a composite semiquantitative scale. This represented a modification of the previously reported scoring systems standardized by our group (13). Specifically, ground-glass opacities (GGO) (alveolar score, AS), consolidations (CONS), and reticulations (interstitial score, IS) were analyzed. For each lung lobe, the two radiologists assessed the extent of AS, CONS, and IS using a scale from 0 to 100 and estimated extent to the nearest 2%. The result was expressed as the mean value of the five lobes in AS, CONS, and IS. The level of interobserver agreement was obtained for each patient as a mean of 5 lobes and for each radiological abnormality (AS, CONS, and IS) and expressed as Cohen's *k* value. Disagreement between radiologists was resolved by consensus.

### Statistical Analysis

Categorical variables were described as absolute (*n*) and relative values (%), whereas continuous variables were described as median and range. To compare demographic and clinical data between REC and NOT-REC patients, chi-square test and Fisher's exact test (*n* < 5) for categorical variables and Mann–Whitney U tests for continuous variables were used, as appropriate.

To compare radiological scores at HRCT_1_ in NOT-REC patients, Mann–Whitney U test for continuous variables was used, whereas Wilcoxon signed-rank test was used to compare radiological scores between HRCT_0_ and HRCT_1_. A univariate logistic regression analysis, followed by a regression model adjusted for gender, pack-years, paO_2_/FiO_2_ at admission, degree of medical care (high or low), and FiO_2_ max, was performed to detect the predictive factors of radiologic sequelae (NOT-REC) at follow-up. All data were analyzed using SPSS Software version 25.0 (US: IBM Corp., New York, NY, USA). *p*-Values < 0.05 were considered statistically significant. The graphs were obtained using the statistical package GraphPad Prism 7.0 (GraphPad Software, Inc., La Jolla, CA, USA).

### Ethics Statement

The study protocol complies with the ethical guidelines of the 1975 Declaration of Helsinki, and in agreement with national regulation on observational studies, it was notified and approved by the local ethics committee (number: 46430/03.08.2020) and the need for patient's informed consent was waived.

## Results

### Clinical Evaluation at Hospital Admission and During Hospitalization

**Figure 1:**
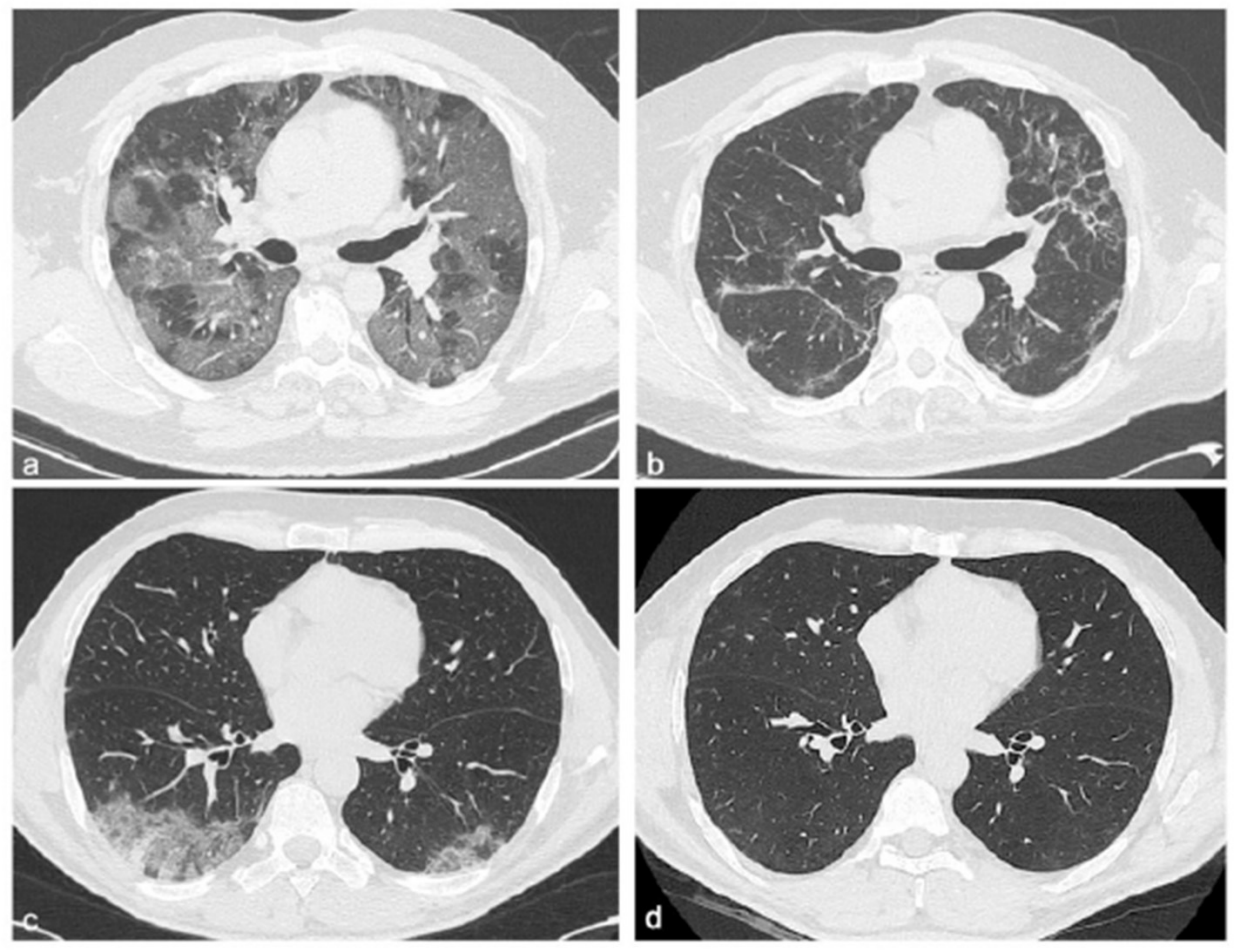
Chest CT features of two patients with COVID-19 pneumonia at different time points: hospitalization and 6 months after discharge. Chest CT images of a 58-year-old male patient with COVID-19, not recovery patient **(a,b)**. The first CT performed at admission shows bilateral areas of ground-glass opacities in a peripheral distribution **(a)**, and after 6 months from discharge, CT shows persistent of interlobular septal thickening with peripheral distribution **(b)**. Chest CT images of a 51-year-old male patient with COVID-19, recovery patient **(c,d)**. The first CT shows, at admission, a small consolidation at the right lower lobe accompanied by ground-glass opacities in both lower lobes **(c)**, and after 6 months from discharge, no residual abnormalities were observed **(d)**.

Two hundred and 20 patients with COVID-19 pneumonia evaluated at the post-COVID clinic were included in the study (Table 1). A total of 115 patients (52%) were men, with a median age of 59 years (range 19–84) and body mass index (BMI) 26 (18–39). The most prevalent comorbidities were CVDs (*n* = 98, 45%), followed by the chronic respiratory diseases (18%). Based on the presence of radiological sequelae on HRCT performed at follow-up (HRCT_1_), 175 (80%) patients were categorized as REC and 45 (20%) as NOT-REC (Figure 1). Baseline demographic and clinical data of REC and NOT-REC patients are summarized in Table 1.

**Table 1:** Baseline demographics and clinical features of the overall population evaluated at post-COVID clinic, and of the two subgroups categorized according to the presence of radiological recovery during the follow-up period.

	**Overall population (*n* = 220)**	**REC (*n* = 175; 80%)**	**NOT—REC (*n* = 45; 20%)**	***P* value**
Male—*n (%)*	115 (52)	86 (49)	29 (64)	0.06
Age at admission—*years*	59 (19–87)	56 (19–87)	66 (35–85)	<0.0001
Smoking history—*pack-years*	0 (0–67)	0 (0–67)	0 (0–60)	0.07
Current—*n (%)*	15 (7)	10 (6)	5 (11)	0.20
Former—*n (%)*	70 (32)	54 (31)	16 (36)	0.54
Nonsmokers—*n (%)*	135 (61)	111 (63)	24 (53)	0.21
BMI—(kg/m^2^)	26 (18–39)	27 (18–39)	26 (21–35)	0.35
Cardiovascular diseases—*n* (%)	98 (45)	72 (41)	26 (58)	0.04
Respiratory diseases—*n* (%)	39 (18)	30 (17)	9 (20)	0.65
Autoimmune diseases—*n* (%)	36 (16)	25 (14)	11 (24)	0.10
Metabolic diseases—*n* (%)	102 (4)	78 (45)	24 (53)	0.29
Oncologic diseases—*n* (%)	25 (11)	17 (8)	8 (18)	0.12
PaO_2_ / FiO_2_ at admission	314 (33–543)	318 (33–543)	233 (40–424)	0.04
FiO_2_max during hospitalization—%	28 (21–100)	27 (21–100)	45 (21–100)	<0.0001
Hospitalization—days	9 (0–75)	8 (1–52)	16 (0–75)	<0.0001
Low degree of care—*n* (%)	163 (74)	138 (79)	25 (56)	0.002
High degree of care—*n* (%)	57 (26)	37 (21)	20 (44)	

*Values are expressed as numbers and (%) or median and range, as appropriate. To compare demographics between recovery (REC) and not recovery (NOT-REC), chi-square test and Fisher's t-test (n < 5) for categorical variables and Mann–Whitney t-test for continuous variables were used*.

No differences in sex, smoking history, or BMI were observed between the two groups, with a prevalence of men in NOT-REC compared to REC (64 vs. 49%, respectively). NOT-REC patients were significantly older compared to REC [66 (35–85) vs. 56 (19–87) years; *p* < 0.0001]. CVDs were significantly more frequent in NOT-REC compared to REC [26 (58%) vs. 72 (41%); *p* = 0.04] whereas autoimmune, metabolic, and oncologic diseases did not differ between the two groups. Symptoms before hospital admission were also similar, except for a higher proportion of patients presenting with dyspnea in NOT-REC compared to REC group [33 (73%) vs. 64 (37%); *p* < 0.0001] (Supplementary Table 1).

At hospital admission, NOT-REC had a worse gas exchange with a lower PiO_2_/FiO_2_ ratio than REC [233 (40–424) vs. 318 (33543); *p* = 0.04]. In addition, compared to REC, during hospitalization, NOT-REC required more frequently high-intensity medical care (HIMC) (20, 44 vs. 37, 21%; *p* = 0.002), higher FiO_2_ max [45 (21–100) vs. 27 (21–100); *p* < 0.0001], and longer in-hospital stay [16 (0–75) vs. 8 (1–52) days; *p* < 0.0001].

The majority of patients were admitted during the first SARS-CoV-2 wave when no standardized protocols existed for treatment of hospitalized patients. NOT-REC patients were more frequently treated with hydroxychloroquine (*n* = 37, 82 vs. 111, 63%; *p* = 0.01), antibiotics other than ceftriaxone and azithromycin (*n* = 25, 56 vs. 44, 25%; *p* < 0.0001), remdesevir (*n* = 7, 16 vs. 10, 6%, *p* = 0.02), tocilizumab (*n* = 8, 18 vs. 12, 7%; *p* = 0.02), and steroids (*n* = 27, 60 vs. 74, 42%; *p* = 0.03) compared to REC. Conversely, the two groups did not differ with regard to the use of ceftriaxone, azithromycin, lopinovir/ritonavir, and hyperimmune plasma (Supplementary Table 2). At discharge, a similar proportion of patients in both groups were prescribed steroids.

### Clinical, Functional, and Radiologic Evaluation at Follow-Up

Patients were evaluated at post-COVID clinic at regular 3-month intervals after discharge. At first evaluation, NOT-REC patients presented more frequently a modified Medical Research Council (mMRC) scores of 1 and 2 compared to REC [15 (33%) vs. 22 (13%), *p* = 0.0009 and 7 (16%) vs. 3 (2%), *p* < 0.0001, respectively]. In the overall population, pulmonary function tests (PFTs) revealed a median forced vital capacity (FVC) of 3.40 liters (L) (range 1.40–7.96), 96%pred. and a median total lung capacity (TLC) of 5.36 L (3.63–8.09), 89% pred. within the normal range. Likewise, NOT-REC patients showed preserved lung volumes within normal range (Supplementary Table 3). A number of 32 patients out of 220 (14.5%) had an abnormal diffusing capacity of the lung for carbon monoxide (DLco) at the 6-month follow-up, which occurred in those with persistent interstitial lung abnormalities (NOT-REC patients). At follow-up CT (HRCT_1_), NOT-REC patients presented higher ALV [2.8 (0.0–40.0)] compared to CONS [0.0 (0.0–2.0); *p* < 0.0001] and IS [0.6 (0.0–24.0); *p* < 0.0001] (Supplementary Figure 1). Overall, the interobserver agreement between the two radiologists with regard to change in AS, CONS, and IS was good (Cohen's kappa = 0.79 for AS, k = 0.88 for CONS, and k = 0.81 for IS).

### Longitudinal Evaluation of Radiologic Manifestation: From Hospitalization to Follow-Up

**Figure 2:**
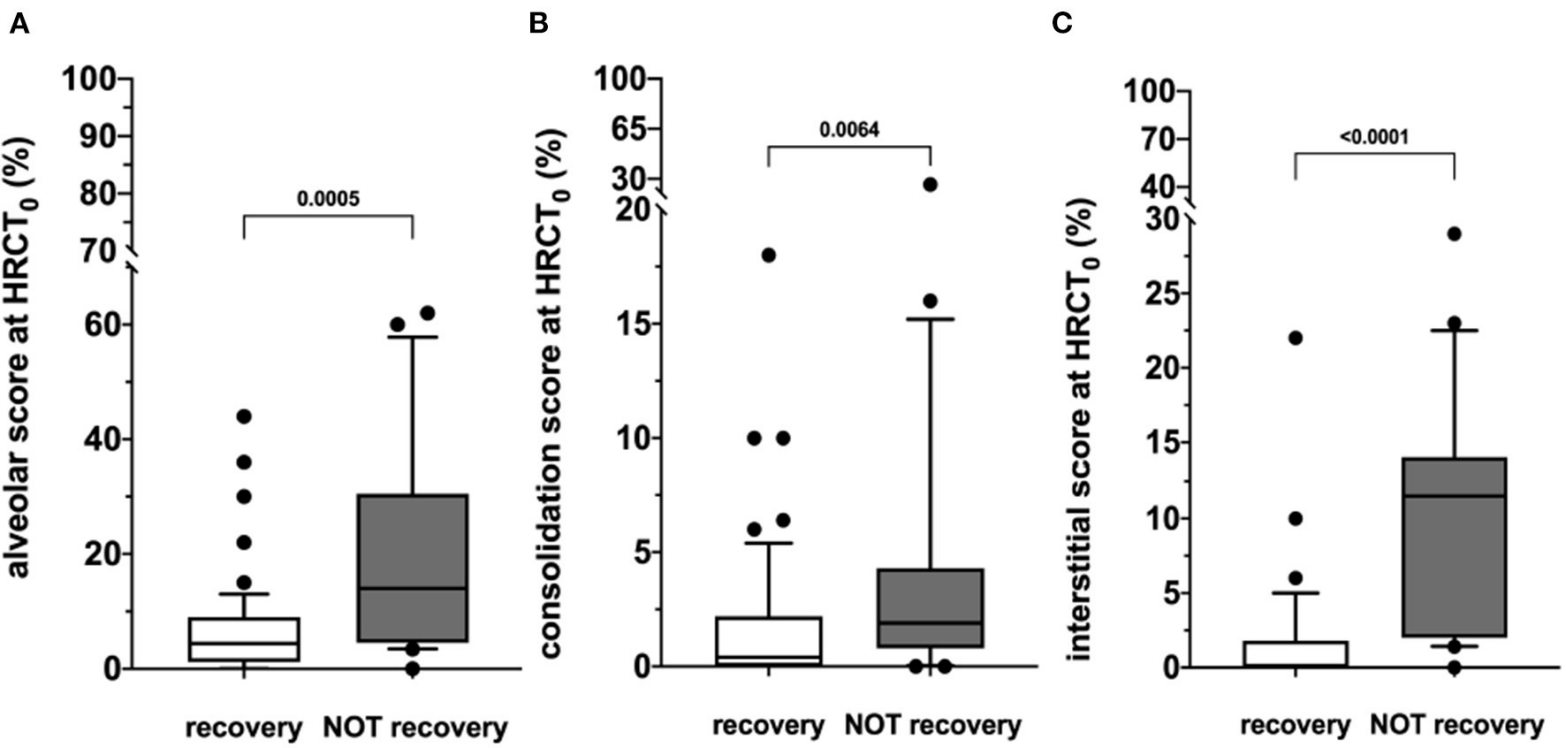
HRCT scores during hospitalization (HRCT0) of the two subgroups categorized according to the presence of radiological recovery [recovery (REC) or NOT-recovery (NOT-REC)] at follow-up period. Horizontal bars represent median values; bottom and top of each box plot 25th and 75th; brackets show 10th and 90th percentiles; and circles represent outliers. White boxes indicate values for recovery group and gray boxes not recovery group. **(A)** ALV [14.0 (0.0–62.0) vs. 4.4 (0.0–44.0); *p* = 0.0005]; **(B)** CONS [1.9 (0.0–26.0 vs. 0.4 (0.0–18.0); *p* = 0.0064]; **(C)** INT [11.5 (0.0–29.0) vs. 0.0 (0.0–22.0); *p* < 0.0001].

**Figure 3:**
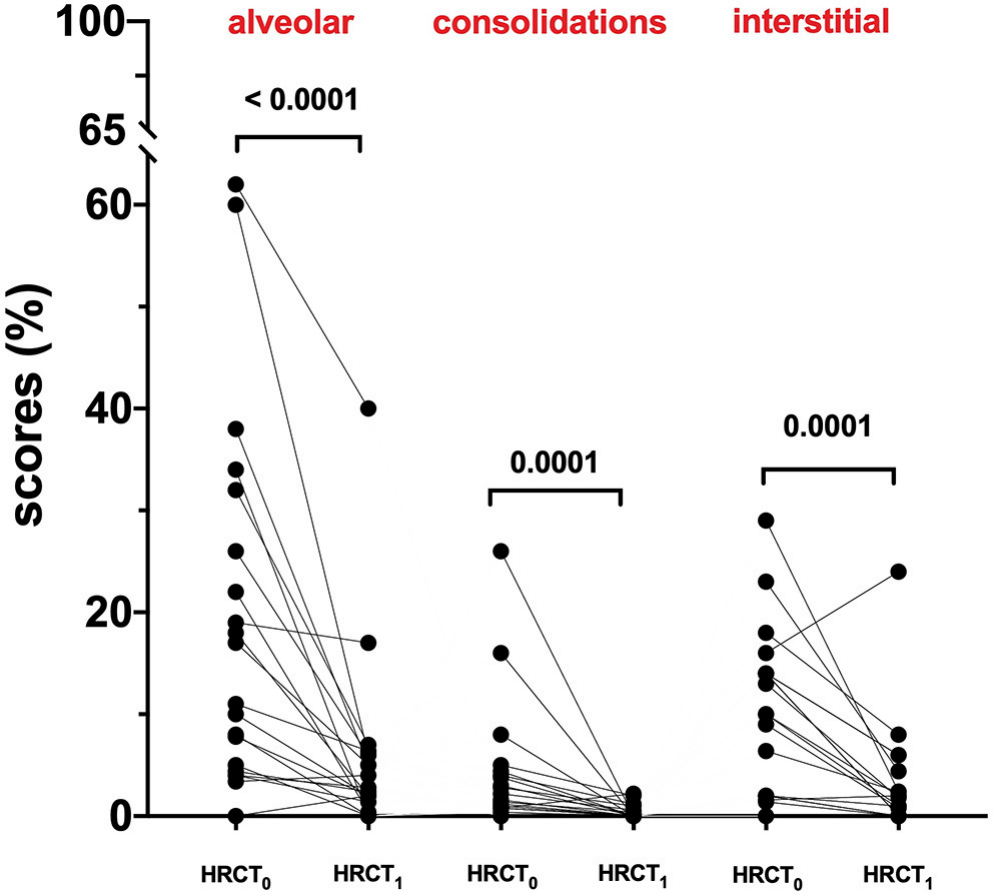
HRCT scores of the not recovery population (NOT-REC) from HRCT0 to HRCT1: ALV. [from 14 (0.0–62.0) to 2.6 (0.0–40.0); *p* < 0.0001], CONS [from 1.9 (0.0–26.0) to 0.0 (0.0–2.2); *p* = 0.0001] and INT [1.5 (0.0–29.0) to 1.4 (0.0–24.0)].

At hospital admission, HRCT (HRCT_0_) was available for 79/220 (36%) patients. ALV [5.0 (0.0–62.0)] was significantly more prevalent compared to CONS [0.8 (0.0–26.0); *p* < 0.0001] and IS [0.8 (0.0–29.0); *p* < 0.0001]. When this patient subgroup was stratified in NOT-REC and REC, NOT-REC patients (*n* = 20) had at hospital admission higher ALV [14.0 (0.0–62.0) vs. 4.4 (0.0–44.0); *p* = 0.0005] (Figure 2A), CONS [1.9 (0.0–26.0 vs. 0.4 (0.0–18.0); *p* = 0.0064] (Figure 2B), and IS [11.5 (0.0–29.0) vs. 0.0 (0.0–22.0); *p* < 0.0001] (Figure 2C) compared to REC patients (*n* = 59) (Table 2). Finally, when comparing HRCT_0_ with HRCT_1_, we observed that in NOT-REC patients, ALV [from 14 (0.0–62.0) to 2.6 (0.0–40.0); *p* < 0.0001], CONS [from 1.9 (0.0–26.0) to 0.0 (0.0–2.2); *p* = 0.0001], and IS [1.5 (0.0–29.0) to 1.4 (0.0–24.0)] decreased significantly (Figure 3).

**Table 2:** HRCT scores during hospitalization (HRCT_0_) of the overall population evaluated at post-COVID clinic and of the two subgroups categorized according to the presence of radiological recovery during the follow-up period.

	**Overall population (*n* = 220)**	**REC (*n* = 175; 80%)**	**NOT—REC (*n* = 45; 20%)**	***p*-value**
**Alveolar score—%**	5.0 (0.0–62)	4.4 (0.0–44.0)	14.0 (0.0–62.0)	0.0005
**Consolidations—%**	0.8 (0.0–26.0)	0.4 (0.0–18.0)	1.9 (0.0–26.0)	0.006
**Interstitial score—%**	0.8 (0.0–29.0)	0.0 (0.0–22.0)	11.5 (0.0–29.0)	<0.0001

*Values are expressed as median and range, as appropriate. To compare HRCT scores at hospitalization (HRCT_0_) between recovery (REC) and not recovery (NOT-REC), Mann–Whitney t-test for continuous variables was used*.

### Prognostic Factors for Radiological Sequelae at Follow-Up

Univariate analysis showed that older age, a prolonged in-hospital stay, a lower PiO2/FiO_2_ at hospital admission, cardiovascular comorbidities, a higher degree of medical care, a higher FiO_2_ max, and higher ALV, CONS, and INT scores at HRCT_0_, not use of hydroxychloroquine, antibiotics other than azithromycin and ceftriaxone, tocilizumab, remdesevir, and systemic steroids are associated with persistent radiological abnormalities at follow-up. Multivariate analysis revealed that CONS [OR: 20.6 (95%CI: 1. −301.2); *p* = 0.02] and IS score [23.0 (1.4–377.2); *p* = 0.02] are independent predictors of radiological sequelae at follow-up (Table 3).

**Table 3:** Predictive factors of radiological sequelae at follow-up in patients hospitalized for SARS-COV-2-related pneumonia.

	**Univariate analysis**		**Multivariate analysis**	
	**OR (95% IC)**	***p***	**OR (95% IC)**	***p***
Sex				
Female Male	Ref. 1.87 (0.95—3.69)	- 0.07	- -	- -
Age—years				
<59 ≥59	Ref. 2.99 (1.47–6.08)	- 0.002	Ref. 0.81 (0.10–6.39)	- 0.84
BMI—(kg/m^2^)				
<26 ≥26	Ref. 0.80 (0.41–1.58)	- 0.52	- -	- -
Smoking history–*pack–years*				
*=* 0 >0	Ref. 1.56 (0.79–3.10)	- 0.19	- -	- -
Hospitalization—*days*				
<9 ≥9	Ref. 4.77 (2.15–10.5)	- <0.0001	Ref. 12.77 (0.65–248.8)	- 0.09
PiO_2_/FiO_2_ at admission				
<314 ≥314	Ref. 0.33 (0.13–0.80)	- 0.01	Ref. 1.24 (0.13–11.46)	- 0.84
CVD				
No Yes	Ref. 1.95 (1.00–3.80)	- 0.04	Ref. 1.40 (0.15–12.48)	- 0.76
Respiratory diseases				
No Yes	Ref. 1.20 (0.52–2.77)	- 0.65	- -	- -
Autoimmune diseases				
No Yes	Ref. 1.94 (0.87–4.32)	- 0.11	- -	- -
Metabolic diseases				
No Yes	Ref. 1.42 (0.73–2.74)	- 0.29	- -	- -
Oncologic diseases				
No Yes	Ref. 2.01 (0.80–5.01)	- 0.13	- -	- -
Degree of care				
Low High	Ref. 2.98 (1.49–5.95)	- 0.002	Ref. 1.35 (0.13–13.12)	- 0.79
FiO_2_ max—%				
<28 ≥28	Ref. 3.25 (1.54–6.80)	- 0.002	Ref. 1.01 (0.07–16.2)	- 0.99
Alveolar score HRCT0—%				
<7 ≥7	Ref. 4.0 (1.33–11.98)	- 0.01	Ref. 0.74 (0.09–5.99)	- 0.78
Consolidations HRCT0—%				
<0.8 ≥0.8	Ref. 6.29 (1.66–23.87)	- 0.007	Ref. 20.6 (1.40–301.2)	- 0.02
Interstitial score HRCT0—%				
<1.4 ≥1.4	Ref. 41.2 (5.1–331.8)	- <0.0001	Ref. 23.0 (1.40–377.2)	- 0.02
Hidroxicloroquina				
Yes No	Ref 2.66 (1.17–6.07)	0.02	Ref 1.26 (0.18–8.82)	0.80
Azithromycin				
Yes No	Ref. 0.76 (0.39–1.47)	- 0.41	- -	- -
Ceftriaxone				
Yes No	Ref. 1.74 (0.89–3.40)	- 0.10	- -	- -
Other antibiotics				
Yes No	Ref. 3.72 (1.88–7.34)	- <0.0001	Ref. 4.87 (0.52–45.7)	- 0.16
Lopinovir/Ritonavir				
Yes No	Ref. 1.49 (0.75–2.94)	- 0.24	- -	- -
Remdesevir				
Yes No	Ref. 3.03 (1.08–8.49)	- 0.03	Ref. 12.5 (0.41–3.85)	- 0.14
Glutathione				
Yes No	Ref. 0.22 (0.09–1.75)	- 0.15	- -	- -
Tocilizumab				
Yes No	Ref. 2.93 (1.12–7.69)	- 0.02	Ref. 0.6 (0.03–11.1)	- 0.73
Plasma				
Yes No	Ref. 1.49 (0.37–5.86)	- 0.56	- -	- -
Steroids during hospitalization				
Yes No	Ref. 2.04 (1.05–3.99)	- 0.03	Ref. 1.04 (0.09 – 11.6)	- 0.97

*Values are expressed as OR (95%CI). Logistic regression analysis was used to determine the relationship of clinical data with radiological sequelae at follow-up*.

Finally, on multivariate analysis adjusted for gender, pack-years, PiO_2_/FiO_2_ ratio at admission, degree of care (high or low), and FiO_2_ max, both CONS and IS at HRCT_0_ are independent predictors of radiological sequelae at follow-up with an OR of 14.87 (95% CI: 1.25–175.8; *p* = 0.03) and 28.9 (95% CI: 2.17–386.6; *p* = 0.01), respectively (Table 4).

**Table 4:** Multivariate analysis for factors independently associated with radiological sequelae at follow-up in patients hospitalized for SARS-COV-2-related pneumonia.

	Multivariate analysis^*^	
	**OR (95% IC)**	***p***
Alveolar score HRCT0—%		
<7 ≥7	Ref. 1.80 (0.39-−8.20)	- −0.44
Consolidations HRCT0—%		
<0.8 ≥0.8	Ref. 14.87 (1.25-−175.8)	- −0.03
Interstitial score HRCT0—%		
<1.4 ≥1.4	Ref. 28.9 (2.17-−386.6)	- -**0.01**

*Values are expressed as OR (95%CI). Univariate and multivariate-adjusted odds ratio for radiological NOT recovery according to radiological patterns during hospitalization (HRCT_0_)*.

* *Adjusted for gender, pack-years, PiO_2_/FiO_2_ ratio at admission, degree of care (high or low), FiO_2_ max*.

## Discussion

In our study, we demonstrated that only a significant minority of patients hospitalized for COVID-19 pneumonia has persistent radiological abnormalities at follow-up. Patients who did not recover are mainly elder men, with a more severe gas exchange impairment at hospital admission and a more severe clinical course during hospitalization. Interestingly, the presence of reticulation and consolidation at admission was predictive of persistent interstitial changes at follow-up.

To date, few studies have reported on the follow-up of patients hospitalized for COVID-19 pneumonia (5, 6). Different approaches based on disease severity have been proposed with the aim to standardize patients' follow-up. Specifically, the British Thoracic Society guidelines for management of post-COVID-19 syndrome distinguished patients with severe pneumonia requiring intensive care from patients with mild-to-moderate pneumonia treated in a medical ward or at home (4). However, it is becoming increasingly clear that radiological changes following COVID-19 pneumonia do not resolve completely in a large minority of patients (5, 15). Some studies have started to use CT to assess the presence of long-term lung abnormalities. A recent work from the Chongqing University Three Gorges Hospital evaluated 41 patients and showed that in most patients, the chest CT lesions were no longer present at 7 months after discharge, whereas older patients with severe comorbidities were more prone to develop fibrosis. (16). From the Wuhan cohort, Han and colleagues investigated 114 patients with severe pneumonia according to the WHO criteria (12) and observed fibrotic changes in one-third of them at the 6-month follow-up. Of note, on multivariate analysis, they found that a higher baseline/initial CT lung involvement score (>18 in a score of 25) was independently associated with fibrotic-like changes in the lung (12). Huang and colleagues conducted a cohort study that included 353 patients who were enrolled between January and May 2020 who underwent HRCT at follow-up after discharge. They found that more than 50% of the patients had residual lung abnormalities. Moreover, they found that disease severity in the acute phase was independently associated with the percentage change of CT score in a multivariable analysis (17).

In our hospital, the first patients with COVID-19 pneumonia were admitted in February 2020 and were evaluated in the post-COVID clinic in June 2020. We enrolled prospectively patients diagnosed with COVID-19 pneumonia according to the WHO criteria. Two hundred and 20 patients were evaluated at 3 months after discharge and every 3 months thereafter, according to the current guidelines (8). We found that as many as 20% of our entire patient population had radiological pulmonary sequelae at follow-up. This percentage is lower than that observed in previous studies (12, 17), but our patients' population has been followed up for a longer period of time, thus allowing non-fibrotic pulmonary abnormalities to clear. Patients who did not recover (NOT-REC) were older, mostly men and with worse disease impairment both at admission and during hospitalization compared to patients without radiological sequelae at follow-up. Specifically, NOT-REC patients had a lower PiO_2_/FiO_2_ ratio at admission and a more severe clinical course. Moreover, NOT-REC patients who required higher maximal FiO_2_ during hospital stay were more often treated in a high-intensive care setting and required a longer in-hospital stay, consistent with the findings from the Wuhan cohort (17). Furthermore, we have shown that, in NOT-REC patients, the HRCT performed at hospital admission is more likely to display ground-glass opacities, consolidations, and reticulation. These data suggest that the risk of pulmonary sequelae may be related to the severity of the acute illness and to the intensity of care needed. This is in line with the hypothesis that a cytokine storm might contribute to the pathogenesis of COVID-19 whereas its severity is associated with poor outcomes (18). However, mechanical ventilation and ventilator-induced lung injury, and high-flow oxygen therapy might also have contributed to the development of fibrotic-like changes (19, 20).

The primary aim of our study was to identify predictors of radiological sequelae following COVID-19 pneumonia. Whereas on univariate analysis age, prolonged in-hospital stay, lower PiO2/FiO_2_ at hospital admission, cardiovascular comorbidities, higher intensity of medical care, and higher FiO_2_ max, not using hydroxychloroquine, antibiotics other than azithromycin and ceftriaxone, tocilizumab, remdesevir, or systemic steroids were significantly associated with the presence of interstitial changes during follow-up, we found that higher CONS [OR: 20.6 (95%CI: 1.4–301.2); *p* = 0.02] and IS [23.0 (1.4–377.2); *p* = 0.02] at hospitalization were the only variables independently associated with the persistence of fibrotic changes at follow-up in multivariate analysis. In particular, this latter observation is consistent with that of Han and colleagues who found that a more extensive baseline or initial CT lung involvement was independently associated with permanent fibrotic-like changes in the lung (12). Additionally, the higher amount of consolidation and reticulation at admission remained significantly associated with persistent radiological abnormalities when adjusted for gender, pack-years of smoking, and PiO_2_/FiO_2_ ratio. However, it remains uncertain whether the fibrotic-like changes we observed represent irreversible pulmonary fibrosis, and further monitoring is warranted to answer this question.

The findings of our study should be interpreted in light of some limitations. First, this is a single-center study; however, it is among the first to analyze HRCT changes over time in a large population of patients hospitalized for COVID-19 pneumonia. In addition, we included a large proportion of patients with severe COVID-19, who are at higher risk of developing persistent lung disease. Second, the CT scan at hospital admission was available for only a subset of patients; however, the aim of our study was to characterize the radiological changes occurring over time as previously done in idiopathic pulmonary fibrosis (21) and to identify predictors of persistent radiological abnormalities.

In conclusion, in our study, about 20% of patients with COVID-19 pneumonia had radiological sequelae at follow-up. Patients who did not fully recover showed a more severe impairment at hospital admission and during hospitalization. Moreover, the presence of reticulation and consolidation on the initial chest CT is predictive of persistent radiological interstitial changes at follow-up.

## Data Availability Statement

The raw data supporting the conclusions of this article will be made available by the authors, without undue reservation.

## Ethics Statement

The studies involving human participants were reviewed and approved by Ethics Committee of the University Hospital of Padova, *via* Niccolò Giustiniani n.2, 35128 Padova (nr.: 46430/03.08.2020). The patients/participants provided their written informed consent to participate in this study.

## Author Contributions

EB, EC, and PS contributed in conceptualization, writing, reviewing and editing, and supervision. EB and EC performed writing original draft—preparation, visualization, and investigation. EC, NB, CG, GC, and AG provided resources and conducted investigation. SP, GC, DL, and AF performed data curation. EB, PS, AC, and MS contributed in resources, visualization, and supervision. All authors have written, read, and approved the final version of the manuscript.

## Conflict of Interest

The authors declare that the research was conducted in the absence of any commercial or financial relationships that could be construed as a potential conflict of interest.

## Publisher's Note

All claims expressed in this article are solely those of the authors and do not necessarily represent those of their affiliated organizations, or those of the publisher, the editors and the reviewers. Any product that may be evaluated in this article, or claim that may be made by its manufacturer, is not guaranteed or endorsed by the publisher.

## Abbreviations

IPF: idiopathic pulmonary fibrosis
FVC: forced vital capacity
HRCT: high-resolution computed tomography
REC: recovered
NOT-REC: not recovered
AS: alveolar score
CONS: consolidations
IS: interstitial score
COVID-19: Coronavirus disease 2019
SARS-CoV-2: severe acute respiratory syndrome coronavirus 2
RT-PCR: real- time polymerase chain reaction
LIMC: low-intensity medical care
HIMC: high-intensity medical care
BMI: body mass index
CVDs: cardiovascular diseases
TLC: total lung capacity
DLCO: diffusing capacity of the lung for carbon monoxide.
