# Supplementary material for: Characteristics and Prognostic Factors of Pulmonary Fibrosis After COVID-19 Pneumonia
Source: Front Med (Lausanne). 2022 Jan 31;8:823600. doi: 10.3389/fmed.2021.823600 (PMC8841677; doi:10.3389/fmed.2021.823600)
Supplement: Supplementary file 1 [file Data_Sheet_1.PDF]

Supplementary Material

Characteristics and prognostic factors of pulmonary fibrosis after COVID-19 pneumonia

Elisabetta Cocconcelli (EC)1†, Nicol Bernardinello (NB)1†, Chiara Giraudo (CG)2, Gioele Castelli (GC)1, Adelaide Giorgino (AG)2, Davide Leoni (DL)3, Simone Petrarulo (SP)1, Anna Ferrari (AF)3, Marina Saetta (MS)1, Annamaria Cattelan (AC)3, Paolo Spagnolo (PS)1, and Elisabetta Balestro (EB)1 *.

1 Respiratory Disease Unit, Department of Cardiac, Thoracic, Vascular Sciences and Public Health, University of Padova and Padova City Hospital, 35128 Padova, Italy Department of Cardiac, Thoracic, Vascular Sciences and Public Health, University of Padova and Padova City Hospital, 35128 Padova, Italy

2 Institute of Radiology, Department of Medicine, University of Padova and Padova City Hospital, 35128 Padova, Italy

3 Division of Infectious and Tropical Diseases, University of Padova and Padova City Hospital, 35128 Padova, Italy

† These two authors share first authorship.

* Corresponding author:

Elisabetta Balestro, M.D. Respiratory Disease Unit, Department of Cardiac, Thoracic, Vascular Sciences and Public Health, University of Padova and Padova City Hospital, 35128 Padova, Italy; telephone: +39 0498213702; e-mail: elisabetta.balestro@aopd.veneto.it

Characteristics and prognostic factors of pulmonary fibrosis after COVID-19 pneumonia

Table 1 Supplement. Symptoms referred before hospitalization of the overall population evaluated at post-COVID clinic, and of the two subgroups categorized according to the presence of radiological recovery during the follow up period.

	**Overall population (n =220)**	**REC (n = 175; 80%)**	**NOT - REC (n = 45; 30%)**	***p* Value**
**Fever –** ***n (%)***	208 (95)	163 (93)	45 (100)	0.07
**Fatigue –** ***n (%)***	89 (40)	75 (43)	14 (31)	0.15
**Dyspnea –** ***n (%)***	97 (44)	64 (37)	33 (73)	**< 0.0001**
**Anosmia/Ageusia –** ***n (%)***	79 (36)	61 (35)	18 (40)	0.52
**Myalgia –** ***n (%)***	47 (21)	38 (22)	9 (20)	0.80
**Alopecia –** ***n (%)***	5 (2)	4 (2)	1 (2)	0.97
**Impaired attention –** ***n (%)***	3 (1)	2 (1)	1 (2)	0.57
**Insomnia –** ***n (%)***	0 (0)	0 (0)	0 (0)	n.a.
**Headache –** ***n (%)***	24 (11)	22 (13)	2 (4)	0.11
**Nausea/Vomiting/Diarrhea –** ***n (%)***	48 (22)	38 (22)	10 (22)	0.94
**Cough –** ***n (%)***	126 (57)	96 (55)	30 (67)	0.15

Values are expressed as numbers and (%). To compare symptoms frequencies between recovery (REC) and not recovery (NOT-REC), Chi square test and Fisher t test ($n < 5$) for categorical variables were used.

Table 2 Supplement. Treatment during hospitalization of the overall population evaluated at post-COVID Clinic, and of the two subgroups categorized according to the presence of radiological recovery during the follow up period.

	**Overall population (n =220)**	**REC (n = 175; 80%)**	**NOT - REC (n = 45; 20%)**	***p* Value**
**Azithromycin – n (%)**	129 (59)	105 (60)	24 (53)	0.41
**Hidroxicloroquina - n (%)**	148 (67)	111 (63)	37 (82)	**0.01**
**Ceftriaxone – n (%)**	75 (34)	55 (31)	20 (44)	0.10
**Other antibiotics – n (%)**	69 (31)	44 (25)	25 (56)	**< 0.0001**
**Lopinovir/Ritonavir – n (%)**	72 (33)	54 (31)	18 (40)	0.24
**Rendesevir – n (%)**	17 (8)	10 (6)	7 (16)	**0.02**
**Glutathione – n (%)**	18 (8)	17 (10)	1 (2)	**0.002**
**Tocilizumab – n (%)**	20 (9)	12 (7)	8 (18)	**0.02**
**Heparin - n (%)**	143 (65)	115 (66)	28 (62)	0.66
**Plasma – n (%)**	11 (5)	8 (5)	3 (7)	0.56
**Steroids during hospitalization – n (%)**	101 (46)	74 (42)	27 (60)	**0.03**

**Steroids after hospitalization – n (%)**	64 (29)	46 (26)	18 (40)	0.07

Values are expressed as numbers and (%). To compare treatment frequencies between recovery (REC) and not recovery (NOT-REC), Chi square test and Fisher t test (n < 5) for categorical variables were used.

Table 3 Supplement. Pulmonary function tests of the overall population evaluated at post-COVID Clinic, and of the two subgroups categorized according to the presence of radiological recovery during the follow up period.

	**Overall population (n =220)**	**REC (n = 175; 80%)**	**NOT - REC (n = 45; 20%)**	***p* Value**
**FVC - liters**	3.40 (1.40 – 7.96)	3.42 (1.40 – 7.96)	3.48 (1.83 – 4.84)	0.66
**FVC - % pred.**	96 (50 - 137)	98 (50 - 137)	93 (68 - 131)	0.25
**FEV1 - liters**	2.88 (0.88 – 6.11)	2.88 (0.88 – 6.11)	2.98 (1.65 – 4.03)	0.82
**FEV1 - % pred.**	100 (46 - 146)	100 (46 - 136)	99 (74 - 146)	0.40
**TLC - liters**	5.36 (3.63 – 8.09)	5.36 (3.81 – 8.09)	5.36 (3.63 – 7.75)	0.78
**TLC - % pred.**	89 (59 - 117)	88 (68 - 117)	89 (59 - 116)	0.67

Values are expressed as median and range. To compare functional parameters between recovery (REC) and not recovery (NOT-REC), Mann Whitney U test for continuous variables was used. FVC = forced vital capacity; FEV1 = Forced expiratory volume in 1 second; TLC = total lung capacity.

Figure 1

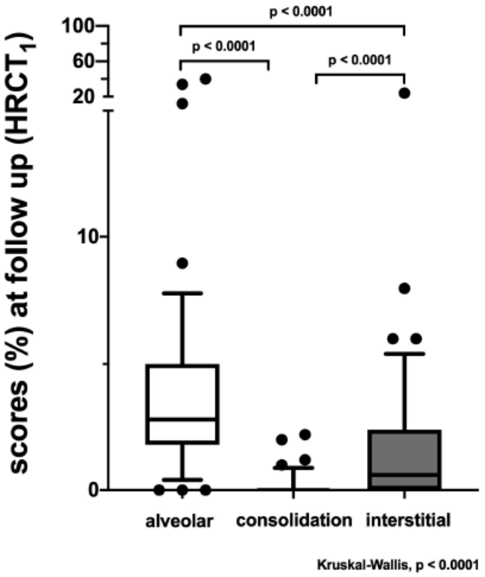

Figure 1 Supplement. HRCT scores at follow up evaluation of the not recovery (NOT-REC) population. Horizontal bars represent median values; bottom and top of each box plot 25th and 75th; brackets show 10th and 90th percentiles; and circles represent outliers. White boxes indicate values for alveolar score and grey boxes for interstitial score. At follow up, NOT-REC patients presented higher ALV [2.8 (0.0 – 40.0)] compared to CONS [0.0 (0.0 – 2.0); $p < 0.0001$] and IS [0.6 (0.0 – 24.0); $p < 0.0001$].
